# Point-of-care ultrasonography and liberation from mechanical ventilation

**DOI:** 10.1186/s13054-017-1649-6

**Published:** 2017-03-16

**Authors:** Gentle Sunder Shrestha

**Affiliations:** Department of Anaesthesiology, Critical Care Unit, Tribhuvan University Teaching Hospital, Maharajgunj, Kathmandu, Nepal

Safe liberation from mechanical ventilation remains a challenge for the critical care physician. Undue prolongation of mechanical ventilation or extubation failure followed by re-intubation is associated with negative outcome. Recent clinical practice guidelines suggest using pressure augmentation during a spontaneous breathing trial (SBT), which would result in more successful SBTs and higher extubation rates. However, despite pressure augmentation, the extubation success rate was only 75.4% [1], reflecting the significant limitation of the SBT to predict extubation success. Moreover, other predictors of weaning success like the Rapid Shallow Breathing Index (RSBI), respiratory system compliance and MIP lack significant predictive value. The guidelines also suggest extubation to preventive non-invasive ventilation (NIV) for patients at high risk for extubation failure despite a successful SBT, which again reflects the limitations of the SBT. High-risk patients were defined variably in different studies, further complicating the task of identifying the vulnerable group of patients who would potentially fail extubation [1].

A significant proportion of patients would pass a SBT and would achieve extubation success irrespective of the technique used for the SBT and without the institution of post-extubation NIV. The challenge for clinicians is to identify the group of patients who would fail extubation after a successful SBT, to potentially optimize the patient before extubation and to identify those patients who would benefit from preventive NIV following extubation.

Change in lung aeration during a SBT can be quantified at the bedside using the validated lung ultrasound score. The scores calculated before and at the end of a SBT can help to detect the extent of aeration loss, which correlates with extubation failure. This can potentially identify the patients who would benefit from preventive NIV following extubation. Similarly, the presence of LV diastolic dysfunction and an increase in LV filling pressure during a SBT can predict extubation failure [2].

Bedside ultrasonography can evaluate diaphragmatic performance in the form of diaphragmatic excursion during inspiration and thickening of the diaphragmatic muscle during inspiration at the zone of apposition. Diaphragmatic dysfunction, as diagnosed by bedside ultrasonography, has been shown to correlate with longer weaning time and higher possibility of extubation failure. Ultrasonographic indices may be equivalent to or better than the RSBI to predict extubation failure [3, 4].

Larger prospective studies need to be conducted to validate and strengthen the existing evidence regarding the use of point-of-care ultrasonography to facilitate safe liberation from mechanical ventilation. Combining the bedside ultrasonography of the lung, the heart and the diaphragm with clinical indices like the RSBI may help to derive a composite index with higher predictive capability.

## Abbreviations

LV: Left ventricular
MIP: Maximal inspiratory pressure
NIV: Non-invasive ventilation
RSBI: Rapid Shallow Breathing Index
SBT: Spontaneous breathing trial
